# Adaptive Evolution in the Glucose Transporter 4 Gene *Slc2a4* in Old World Fruit Bats (Family: Pteropodidae)

**DOI:** 10.1371/journal.pone.0033197

**Published:** 2012-04-06

**Authors:** Bin Shen, Xiuqun Han, Junpeng Zhang, Stephen J. Rossiter, Shuyi Zhang

**Affiliations:** 1 Institute of Molecular Ecology and Evolution, Institutes for Advanced Interdisciplinary Research, East China Normal University, Shanghai, China; 2 School of Biological and Chemical Sciences, Queen Mary University of London, London, United Kingdom; University of Western Ontario, Canada

## Abstract

Frugivorous and nectarivorous bats are able to ingest large quantities of sugar in a short time span while avoiding the potentially adverse side-effects of elevated blood glucose. The glucose transporter 4 protein (GLUT4) encoded by the *Slc2a4* gene plays a critical role in transmembrane skeletal muscle glucose uptake and thus glucose homeostasis. To test whether the *Slc2a4* gene has undergone adaptive evolution in bats with carbohydrate-rich diets in relation to their insect-eating sister taxa, we sequenced the coding region of the *Slc2a4* gene in a number of bat species, including four Old World fruit bats (Pteropodidae) and three New World fruit bats (Phyllostomidae). Our molecular evolutionary analyses revealed evidence that *Slc2a4* has undergone a change in selection pressure in Old World fruit bats with 11 amino acid substitutions detected on the ancestral branch, whereas, no positive selection was detected in the New World fruit bats. We noted that in the former group, amino acid replacements were biased towards either Serine or Isoleucine, and, of the 11 changes, six were specific to Old World fruit bats (A133S, A164S, V377F, V386I, V441I and G459S). Our study presents preliminary evidence that the *Slc2a4* gene has undergone adaptive changes in Old World fruit bats in relation to their ability to meet the demands of a high sugar diet.

## Introduction

High acute blood glucose concentration causes severe physiological dysfunction and even death [1], [2], [3]. To maintain glucose homeostasis, and thus avoid potential toxicity, the blood glucose concentration is maintained within narrow limits by an inter-play between tissue glucose uptake, hepatic glucose production and insulin production [4]. In the first of these regulatory mechanisms, it is skeletal muscle that contributes most to the removal of excess glucose from circulation [4], [5], a process that is mediated by the transmembrane glucose transporter 4 protein (GLUT4) [5]. GLUT4 (encoded by the *Slc2a4* gene) is a family member of sugar transporter proteins that is highly expressed in skeletal muscle, heart and adipose tissue [4]. In response to stimulation by insulin, exercise or contraction, GLUT4 is translocated from intracellular compartments to the plasma membrane of adipocytes and muscle, where it mediates glucose uptake [3], [4], [6]. Studies of transgenic mice revealed that GLUT4 plays a pivotal role in the regulation of skeletal muscle glucose uptake and maintenance of glucose homeostasis [7], [8].

Old World fruit bats (OWFBs) (Pteropodidae) and New World fruit bats (NWFBs) (Phyllostomidae) have independently evolved a carbohydrate-rich diet comprising mostly fruit and/or nectar [9]. To meet the high energy demands of powered flight, while relying on an often highly ephemeral food source, these bats must frequently ingest large quantities of sugar in a short time span [10], [11]. It has recently been shown that the majority of glucose is absorbed via a passive paracellular pathway in the intestinal tract [12], [13], [14]. At the same time, this has raised the issue of how fruit eating bats ingest sugars rapidly and efficiently while maintaining normal glycaemia.

To date, many efforts have been made to elucidate the means by which fruit bats regulate their blood glucose, with several studies identifying physiological and biochemical adaptations. Michelmore et al. (1998) showed that the pancreas of the Old World frugivorous bat *Rousettus aegyptiacus* contained an unusually large volume (9.1%) of endocrine tissue [15]. More recently, Protzek et al. (2010) found that the pancreas of the New World frugivorous bat *Artibeus lituratus* contained a large population of β-cells, and that this species showed elevated sensitivity to glucose and insulin [16]. In addition to these assumed anatomical specializations, biochemical research on New World nectarivorous bat, *Glossophaga soricina* has shown that high skeletal muscle enzymatic flux capacities in this species are similar to those of hummingbirds [17], and it has been suggested that *G. soricina* has evolved a mechanism of high-activity to balance blood glucose and thus avoid hyperglycemia after excess sugar consumption [18]. Several studies have also shown that both OWFBs (e.g. *R. aegyptiacus*) and NWFBs (e.g. *G. soricina*) fuel their metabolic needs exclusively with exogenous sugars rather than endogenous products [19], [20], [21].

Physiological and biochemical adaptations for glucose metabolism in bats should not only require the effective regulation of plasma glucose levels, but in addition the efficient transportation of blood glucose to high energy demanding organs (e.g. flight muscles). Thus, it is reasonable to suspect that key genes involved in carbohydrate metabolism and blood glucose regulation may have been targets of molecular adaptation in the dietary switches seen in fruit and nectar eating bats.

Considering the critical role of GLUT4 in the regulation of skeletal muscle glucose uptake and maintenance of glucose homeostasis, we speculated that the *Slc2a4* gene will have undergone adaptive evolution coincident with the switch in diet in the ancestral OWFBs and NWFBs. To test our hypothesis, we obtained the coding region of *Slc2a4* from 16 bat species (including four OWFBs species and three NWFBs species), and assessed the molecular evolution of this gene in bats and other mammals.

## Materials and Methods

### Ethics Statement

Our procedures involving animals were in accordance with the guidelines of Regulations for the Administration of Laboratory Animals (Decree No. 2 of the State Science and Technology Commission of the People's Republic of China on November 14, 1988).

### Taxonomic Coverage

We studied the diversity and evolution of the *Slc2a4* gene in 16 bat species. From the suborder Yinpterochiroptera, we included four Old World fruit bats: *Pteropus vampyrus*, *Cynopterus sphinx*, *Eonycteris spelaea*, and *Rousettus leschenaultii* (family Pteropodidae). Also from this clade we sequenced three insectivorous bat species from sister families to the Old World fruit bats: *Rhinolophus ferrumequinum* (Rhinolophidae), *Hipposideros pratti* and *H. armiger* (Hipposideridae). From the other main suborder (Yangochiroptera) we included the three New World fruit bats *Leptonycteris yerbabuenae*, *Artibeus lituratus* and *A. jamaicensis* (Phyllostomidae) and six insectivorous species representing four families: *Taphozous melanopogon* (Emballonuridae), *Tadarida brasiliensis* (Molossidae), *Scotophilus kuhlii*, *Myotis ricketti* and *M. lucifugus* (Vespertilionidae) and *Mormoops megalophylla* (Mormoopidae). Of these the latter family is sister to the New World fruit bats. All new *Slc2a4* sequences were deposited to GenBank and accession numbers are JN695651–JN695665.

We also incorporated available published sequences in our analyses; from Ensembl we obtained the *Slc2a4* sequence of the insectivorous bat species *M. lucifugus*, and from GenBank we obtained sequences form the following seven mammal species: *Homo sapiens* (NM_001042), *Mus musculus* (NM_009204), *Rattus norvegicus* (NM_012751), *Equus caballus* (NM_001081866), *Bos taurus* (NM_174604), *Sus scrofa* (NM_001128433), *Canis familiaris* (NM_001159327). Non-bats provided greater phylogenetic coverage and thus more power for detecting selection. Details of all species, sampling localities, and their corresponding *Slc2a4* accession numbers are listed in Table S1.

### Isolation, Amplification and Sequencing

We isolated genomic DNA using DNeasy Blood & Tissue Kit (Qiagen) from wing membrane biopsies from six species (see Table S2) and designed primers to amplify four separate sections of *Slc2a4* gene (first section for exons 1–2, second for exons 3–7, third for exons 8–9 and fourth for exons 10–11) (Table S2). For the remaining nine species, we isolated total RNA from pectoral muscle tissue (stored at −80°C) of euthanized bats using Trizol reagent (Invitrogen). Following the standard protocol, 5 ug total RNA was reverse transcribed to cDNA by SuperScript™ III Reverse Transcriptase kit (Invitrogen). Primers were designed to amplify the coding sequences of *Slc2a4* (see Table S2). For both genomic DNA and pectoral muscle cDNA, Polymerase Chain Reactions (PCR) were conducted using Premix Ex Taq™ (TaKaRa) with the following conditions: denaturation at 95°C for 5 min, 32 amplification cycles [95°C for 30 s, annealing temperature (see Table S2) for 30 s, 72°C for 1∼2 min (depending upon the target length)], and a final extension at 72°C for 10 min. All PCR products were isolated using 1% agarose gels and purified with Gel Extraction Kits (Qiagen), ligated into pGEM-T easy vector (Promega), cloned and sequenced using the Terminator kits (Applied Biosystems) on an ABI 3730 DNA sequencer.

### Phylogenetic Reconstruction

The nucleotide sequences of 23 species were aligned using ClustalX [22] and checked for accuracy by eye, and coding sequences were translated to amino acids using MEGA4 [23]. A Bayesian phylogenetic tree was reconstructed based on aligned nucleotide sequences using MrBayes 3.1.2 [24] with the TPM2uf+Γ nucleotide substitution model selected by jModelTest0.1 [25]. For the Bayesian analysis, we performed 10,000,000 generations of MCMC and sampled every 100 generations, with the first 2,000,000 generations discarded as burn-in. All other options and priors were default settings of MrBayes 3.1.2 software. The standard deviations of split frequencies were stably below 0.01 after 2,000,000 generations of MCMC performances.

### Molecular Evolution Analyses

Before undertaking analyses of molecular evolution, we first tested for evidence of recombination in our dataset, which can lead to the erroneous detection of positive selection. We used GARD [26] implemented in the package HyPhy [27] to estimate the number of statistically supported recombination breakpoints.

For selection tests, we used a phylogenetic tree topology based on the accepted species relationships [28], [29], [30]. To test for positive selection in *Slc2a4*, we derived maximum-likelihood estimates of the rate of nonsynonymous substitutions (d_N_) and synonymous substitutions (d_S_) using PAML CODEML [31].

We first undertook branch models using two-ratio models, in which the d_N_/d_S_ ratio (termed as omega or ω) was allowed to vary between the background and foreground. Separate models were undertaken with the foreground branch set as the ancestral branch leading to OWFBs and to NWFBs. In both cases, the one-ratio model in which ω was fixed among all branches served as the null hypothesis of no branch-wise change in selection pressure. A three-ratio model in which different d_N_/d_S_ ratios were assumed for the background, the ancestral branch of the OWFBs and the ancestral branch of the NWFBs, was also conducted, and compared to the one-ratio model and the two-ratio models described. We also conducted the free ratio model, which allows the *d*_N_/*d*_S_ ratios to vary among all branches, to explore the overall selection pressures of *Slc2a4* in the 23 species under study [32]. For this, the model parameters were compared to those from the one-ratio model, which served as the null expectation of no variation in selection pressure among lineages.

We also applied the branch-site model A in combination with Bayes empirical Bayes (BEB) estimation, to detect positively selected sites along particular branches [33]. In this model, the phylogeny is divided into foreground and background branches. Four site classes of codons are assumed, of which site class 0 and 1 evolve under purifying selection (0<ω_0_<1) and neutral selection (ω_1_ = 1) respectively throughout the tree. The remaining site classes 2a and 2b evolve under, respectively, purifying and neutral selection on background, but are grouped together and allowed to evolve under positive selection on foreground (ω_2_>1). We applied test 1 and test 2 of this branch-site model [34] to the ancestral branches of OWFBs and NWFBs, in which branch-site model A was the alternative hypothesis. In test 1, the null hypothesis was the M1a (Nearly Neutral) model which assumed two site classes: 0<ω_0_<1 and ω_1_ = 1. While for test 2, the null hypothesis was the modified branch-site model A with ω_2_ fixed as 1. The likelihood ratio tests (LRTs) were used to compare the model fit for alternative and null hypotheses.

Finally, we tested for positive selection among sites across the tree. For this we compared site models M7 versus M8. In this test, the M7 model (beta) was the null model with an assumed beta distribution of ω between 0 and 1, while the M8 model (beta & ω) is the alternative model that has an additional site class of ω>1.

In addition to the methods of PAML, we used two alternative approaches to characterize selection pressures in our dataset, both implemented using the Datamonkey web server (http://www.datamonkey.org/). First we used the GA-Branch method [35], in which branches are assigned to a range of ω classes without *a priori* specification of focal lineages of interest. Second, to examine site-wise variation across branches we applied the random effects branch-site model (Branch-site REL) [36], which might be more robust to errors because it does not enforce a uniform section pressure across all background branches predicted to be not under positive selection.

Ancestral sequences were reconstructed using PAML CODEML package [37], and the amino acid substitutions on each branch were then inferred. In order to determine the lineage-specific amino acid changes, we repeated the ancestral states reconstruction by maximum parsimony method using the software Mesquite 2.74 [38]. To gain information on the likely impact of amino acid substitutions in bats, we used SWISS-MODEL to align the GLUT4 from OWFBs to the better-studied related protein GLUT1 (PDB: 1SUK).

## Results

Our final sequence dataset contained 23 taxa, including four Old World fruit bats (family Pteropodidae) and three New World fruit bats (family Phyllostomidae). The nine remaining bats were all insect-eating species, and covered a wide range of families from across the bat phylogeny. The alignment of *Slc2a4* coding sequences spanned 1530 nucleotides, equating to 510 amino acids, of which 104 were variable in eutherian mammals (Figure S1). The fact that the open reading frame was intact with no detectable stop codons or indels suggested that the *Slc2a4* gene is functional in the species studied.

**Figure 1:**
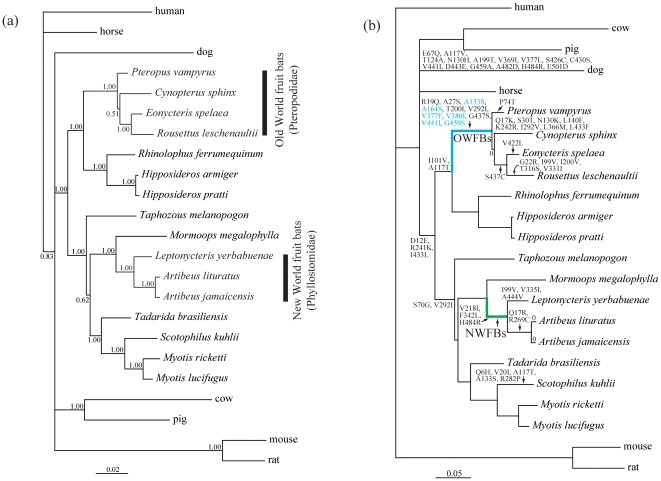
Unconstrained Bayesian phylogenetic tree and species tree. (a) Unconstrained Bayesian phylogenetic tree based on *Slc2a4* coding sequences, under the model of TPM2uf+Γ. Values on the nodes are posterior probabilities. (b) The species tree of 23 mammals based on references 28, 29 and 30. Branch lengths are based on nucleotide substitutions per codon. The blue and green thick lines labeled with ‘OWFBs’ and ‘NWFBs’ represent the ancestral branches of Old World fruit bats and New World fruit bats, respectively. Nonsynonymous amino acid substitutions were mapped onto several selected branches. Six sites on the ancestral branch of Old World fruit bats that had estimated omega values >1 are shown in blue.

Bayesian phylogenetic reconstruction recovered a tree in which the main groupings agreed with the accepted species topology (Figure 1b). Specifically, the Old World fruit bats grouped with the horseshoe bats and their allies to form a well-supported recognised monophyletic clade (suborder Yinpterochiroptera). The remaining lineages were contained within the second major recognised clade, the Yangochiroptera, which included the NWFBs. These bats (all members of the family Phyllostomidae) formed a correct and well-supported monophyletic clade (Bayesian posterior probability = 1.00) that showed a sister relationship with the single representative of the Mormoopidae (Figure 1a). We found no evidence of recombination breakpoints in our dataset, adding further support that the inferred gene tree was valid for molecular genetic analyses.

To assess whether an evolutionary switch to a high carbohydrate diet in OWFBs and NWFBs was associated with positive selection acting on the *Slc2a4* coding sequence, we derived maximum-likelihood estimates of the rates of nonsynonymous substitution (d_N_) and synonymous substitution (d_S_) and undertook a number of branch –wise tests of selection. First, for each of these groups of bats, we constructed a two-ratio branch model (labeled branches in Figure 1b) and, additionally, we used a three-ratio model to estimate simultaneously the ratios for both OWFBs and NWFBs and the background. Results of these branch models are shown in Table 1. Briefly, model comparisons indicated that the two-ratio model with the OWFBs branch designated as the foreground was a significantly better fit than the null (one-ratio) model in which the *d*_N_/*d*_S_ ratio was invariant across all branches [likelihood ratio test (LRT) statistic (2Δ*l*) = 6.03, df = 1, *P* = 0.014] (Table 1). The estimated value of *d*_N_/*d*_S_ on the ancestral OWFBs branch was an order of magnitude greater than that of background (0.257 versus 0.085, respectively) suggesting a change in selection pressure in the evolutionary history of this lineage. However, no such change was found on the ancestral branch of NWFBs, with the one-ratio model not rejected by the two-ratio model (2Δ*l* = 0.02, df = 1, *P* = 0.887) (Table 1). Moreover, a three-ratio model did not fit the data better than the two-ratio model with OWFBs as the foreground (2Δ*l* = 0.04, df = 1, *P* = 0.839). Finally, the free-ratio model test showed that when *d*_N_/*d*_S_ was allowed to vary among branches, the fit was no better than that of a one-ratio model (2Δ*l* = 47.94, df = 42, *P* = 0.244) (Table 1).

**Table 1:** Results of branch model tests of selection pressure on the *Slc2a4* gene in bats.

Model	np	*ℓ*	ω_0_ a	ω_OW_ a	ω_NW_ a	Model Compared	2Δ*ℓ*	*P*
A. One ratio: ω_0_ = ω_OW_ = ω_NW_	45	−7140.94	0.088	= ω_0_	= ω_0_			
B. Two ratios: ω_0_ = ω_OW_, ω_NW_	46	−7140.93	0.088	= ω_0_	**0.098**	B vs. A	0.02	0.887
C. Two ratios: ω_0_ = ω_NW_, ω_OW_	46	−7137.92	0.085	**0.257**	= ω_0_	C vs. A	6.03	0.014
						D vs. A	6.08	0.048
						D vs. B	6.06	0.014
D. Three ratios: ω_0_, ω_OW_, ω_NW_	47	−7137.90	0.085	0.257	0.099	D vs. C	0.04	0.839
E. Free ratio	87	−7116.96	—	—	—	E vs. A	47.94	0.244

a ω_OW_, ω_NW_ and ω_0_, are the ω ratios for branches OWFBs, NWFBs, and other branches, respectively (see Figure 1b).

Application of branch-site models revealed six sites on the ancestral branch of OWFBs with omega values of >1 (133S, 164S, 377F, 386I, 441I and 459S) (Table 2), with branch-site model A found to fit the data better than the M1a Nearly Neutral model (2Δ*l* = 6.85, df = 2, *P* = 0.033; test 1). However, the explicit test of positive selection (test 2) was not significant (2Δ*l* = 1.84, df = 1, *P* = 0.175) (Table 2). Thus we were unable to reject the possibility of relaxed selection, since test 1 of branch-site model A is unable to distinguish relaxed constraints from positive selection [34]. Branch-site models detected no positively selected sites on the ancestral branch of NWFBs, with the null model unable to be rejected over the alternative model in both test 1 and test 2. Other tests of positive selection (M7 versus M8 model comparison), and alternative approaches based on branch (GABranch) and branch-site comparisons (REL) also failed to detect positive selection in both OWFBs and NWFBs (data not shown).

**Table 2:** Results of branch-site model A test for detection of positively selected sites in ancestral branches of Old World fruit bats and New World fruit bats.

Branch-site model	np^a^	Parameters	LRT^b^	*ℓ*	*P*	Sites with elevated omega values^c^
M1a (Nearly Neutral)	46	*p*_0_ = 0.923, *P*_1_ = 0.077, ω_0_ = 0.036, ω_1_ = 1.00		−7024.22		Not allowed
Model A (alternative hypothesis) forOld World fruit bats	48	*P*_0_ = 0.907, *P*_1_ = 0.073, *P*_2a_ = 0.018, *P*_2b_ = 0.001Background: ω_0_ = 0.035, ω_1_ = 1.00, ω_2a_ = 0.035, ω_2b_ = 1.00Foreground: ω_0_ = 0.035, ω_1_ = 1.00, ω_2a_ = 8.765, ω_2b_ = 8.765	Test 1	−7020.79	**0.033**	133S( 0.751), 164S(0.760), 377F( 0.612), 386I( 0.929), 441I( 0.652), 459S( 0.701)
Model A (null hypothesis) forOld World fruit bats	47	*P*_0_ = 0.826, *P*_1_ = 0.066, *P*_2a_ = 0.100, *P*_2b_ = 0.008Background: ω_0_ = 0.035, ω_1_ = 1.00, ω_2a_ = 0.035, ω_2b_ = 1.00Foreground: ω_0_ = 0.035, ω_1_ = 1.00, ω_2a_ = 1.00, ω_2b_ = 1.00	Test 2	−7021.71	**0.175**	Not allowed
Model A (alternative hypothesis) forNew World fruit bats	48	*P*_0_ = 0.923, *P*_1_ = 0.077, *P*_2a_ = 0.00, *P*_2b_ = 0.00Background: ω_0_ = 0.036, ω_1_ = 1.00, ω_2a_ = 0.036, ω_2b_ = 1.00Foreground: ω_0_ = 0.036, ω_1_ = 1.00, ω_2a_ = 1.00, ω_2b_ = 1.00	Test 1	−7024.22	1	Not allowed
Model A (null hypothesis) forNew World fruit bats	47	*P*_0_ = 0.923, *P*_1_ = 0.077, *P*_2a_ = 0.00, *P*_2b_ = 0.00Background: ω_0_ = 0.036, ω_1_ = 1.00, ω_2a_ = 0.036, ω_2b_ = 1.00Foreground: ω_0_ = 0.036, ω_1_ = 1.00, ω_2a_ = 1.00, ω_2b_ = 1.00	Test 2	−7024.22	1	Not allowed

a np, number of parameters.

b LRT, likelihood ratio test.

c Sites with elevated omega values detected by branch-site model A test are referred to *Pteropus vampyrus*. BEB posterior probabilities are shown in parentheses.

**Figure 2:**
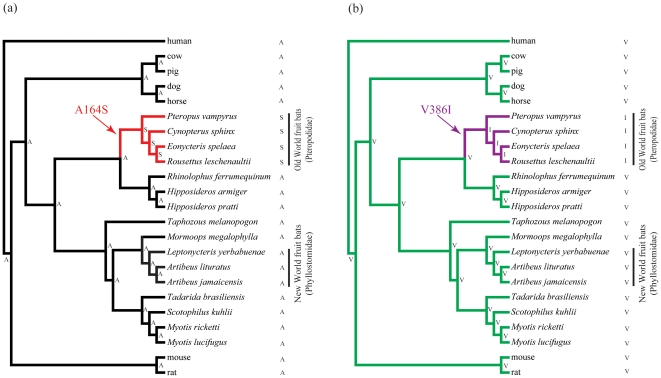
The species tree of 23 mammals with Old World fruit bats specific amino acid replacements were highlighted by ancestral sequence reconstruction using maximum parsimony method. (a) A164S, (b) V386I. Branch lengths are not drawn to scale. The amino acids at positions 164 (A: Ala; S: Ser) and 386 (V: Val; I: Ile) of *Slc2a4* for each interior and exterior node are shown.

**Figure 3:**
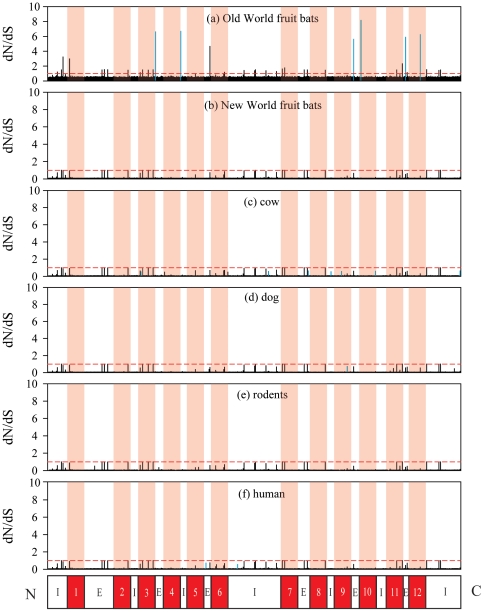
Omega (dN/dS) values for sites along the *Slc2a4* sequence of selected branches. (a) Old World fruit bats, (b) New World fruit bats, (c) cow, (d) dog, (e) rodents and (f) human. The omega value of 1 is indicated by red dash line in each plot. Domains of the GLUT4 protein [4] are shown under the plot: 12 transmembrane domains are colored with red and pink, “I” indicates intracellular domain, “E” indicates extracellular domain. Amino acid sites having BEB posterior probability (>0.5) of being under positive selection are shown in blue.

Examination of reconstructed ancestral sequences revealed a total of 11 amino acid replacements on the ancestral branch of OWFBs, none of which were shared with the NWFBs (Figure 1b). However, of the six sites showing evidence of selection pressure change in the OWFBs (A133S, A164S, V377F, V386I, V441I and G459S), one (A133S) was also detected in *Scotophilus kuhlii*, and three (V377L, V441I and G459A) were seen in the dog (Figure S2). The site with the highest probability of positive selection (386I, BPP = 0.929) was only seen in the Old World fruit bats (Figure 2). The ancestral branches of the Chiroptera, Yinpterochiroptera, Yangochiroptera and NWFBs each contained either two or three amino acid substitutions (Figure 1b). We also calculated the *d*_N_/*d*_S_ values for each amino acid of *Slc2a4* on branches leading to cow, dog, rodents and human, which all exhibited large amount of substitutions, however, no evidence of positive selection was found on these branches (Figure 3).

**Figure 4:**
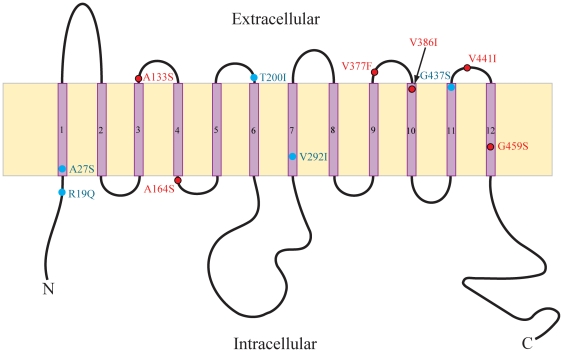
Distribution of 11 amino acid substitutions which occurred on the ancestral branch of Old World fruit bats in the secondary structure of GLUT4 protein with 12 transmembrane domains. Protein structure is adapted from [4]. Six sites with omega values >1 (branch-site model A) are highlighted in red, and the other amino acid substitutions are indicated in blue.

Mapping the 11 amino acid changes in the OWFBs onto the protein secondary structure revealed that four were distributed on extracellular loops (A133S, T200I, V377F and V441I), two within intracellular regions (R19Q and A164S), and the other five within the transmembrane domains (A27S, V292I, V386I, G437S and G459S) (Figure 4). The sites showing evidence of elevated selection pressure were mainly (four of six) located in either extracellular or intracellular loops, extremely close to transmembrane domains (Figure 4).

## Discussion

By comparing the *Slc2a4* gene in frugivorous and nectarivorous bats to their insect-eating sister taxa, we provide some of the first data on molecular adaptation to a sugar rich diet in mammals [39]. We found evidence of a significantly higher value of ω (*d*_N_/*d*_S_) on the ancestral branch of Old World fruit bats (OWFBs) compared to the rest of the tree, indicative of change in selection pressure in *Slc2a4* since the divergence of this lineage from echolocating Yinpterochiroptera. Subsequent analyses revealed six sites with elevated selection pressure.

In spite of this result, based on a branch-site model test of positive selection we were unable to reject the possibility that the ω value in OWFBs had arisen due to relaxed functional constraint, and indeed alternative methods of positive selection proved negative. However, we argue that relaxed evolution at *Slc2a4* in OWFBs is unlikely. This gene is highly conserved across divergent lineages of mammals, including rodents, primates, carnivores and herbivores, a fact that is unsurprising given that the GLUT4 protein product is critical for glucose homeostasis in mammals [4]. Studies of transgenic mice revealed that targeted disruption of GLUT4 in adipocytes and muscle tissues can cause severe insulin resistance and glucose intolerance [7], [8].

If we assume that relaxed selection in the *Slc2a4* gene of OWFBs is indeed implausible, then the best explanation for the significantly high ω in OWFBs is molecular adaptation. Previously it has been pointed out that signals of positive selection can be swamped by a history purifying selection [34], which seems possible given that OWFBs diverged from the other Yinpterochiroptera nearly 60 million years ago [29]. Low sequence divergence in a dataset will also limit the power to detect positive selection [40], [41], [42] and in this context it is interesting to note that our free-ratio model of selection pressure did not fit the data better than a one-ratio model, emphasizing the strong sequence conservation in the *Slc2a4* gene across the taxa studied.

Most of the observed amino acid replacements in the ancestral OWFBs were seen to be concentrated in the external (i.e. intracellular and extracellular) loops of the protein, hinting at some adaptive role in efficient skeletal muscle glucose uptake. Indeed, the inferred change from valine to phenylalanine (V377F) involves the introduction of an extra aromatic ring, might enhance the binding ability of hydrophobic ligands via stacking interactions [43]. Surprisingly, nine of the 11 replacements were to either a serine or an isoleucine, a level of amino acid substitution bias that is not apparent in the other mammal branches (Figure S3 and Table S3). Of these nine, our protein comparisons showed that the residues A164S and V386I in OWFBs are homologous to residues S148 and V370 in the GLUT1 protein, in which they have been implicated both in stabilizing protein packing for facilitation of glucose transport, and in modifying hydrophobic interactions between helices 4–5 and helices 8–10 [44].

In contrast to OWFBs, we found no evidence of elevated ω in their New World counterparts (NWFBs), in which *Slc2a4* was under purifying selection. Moreover, none of the sites found in the OWFBs were shared by the NWFBs. Thus in spite of their convergent eating habits and similar physiological adaptations, there were no detected parallel signatures of selection in this gene, or convergent changes of the sort recently reported in the hearing genes of bats [45], [46], [47], [48]. In this context, it is interesting to note that recent behavioural and physiological data have shown that the New World nectarivorous bat (*Glossophaga soricina*) regulates its blood glucose via high activity behaviour [18], perhaps reducing reliance on other mechanisms of glucose homeostasis such as insulin-stimulated transmembrane glucose uptake. We also cannot rule out the possibility that further adaptive changes underpinning glucose metabolism have occurred in promoter regions rather than in coding sequence [49]. Indeed, recent evidence has suggested that a non-coding region of *Slc2a4* has been the target of natural selection in human populations [50].

Apart from *Slc2a4*, other genes might also have undergone adaptive change in bats (and other mammals) in relation to their diets. For example, it was recently reported that both OWFBs and NWFBs have also undergone adaptive losses of a mitochondrial targeting motif in a key dietary enzyme: AGT (alanine-glyoxylate aminotransferase 1) [39]. As a consequence of this mutation, the AGT enzyme instead accumulates in the peroxisomes, where it breaks down the accumulating plant-derived glyoxylate before it is converted to the toxic oxalate. The same study also showed that AGT has undergone positive selection in the ancestral branch of OWFBs [39]. Similarly, proteins involved in fat metabolism have also been reported to have undergone adaptive changes in species that hibernate [51], [52]. We speculate that as more studies are undertaken, numerous other genes will be seen to have been targets of natural selection during the diversification of mammalian dietary habits.

## Supporting Information

Figure S1 **Alignment of the amino acid sequences of the** ***Slc2a4*** **gene from 23 mammals (only the variable sites are shown).** The species of Old World fruit bats and New World fruit bats are marked in red and blue, respectively.
(TIF)

Figure S2 **The species tree of 23 mammals with Old World fruit bats specific amino acid replacements were highlighted by ancestral sequence reconstruction using maximum parsimony method.** (a) A133S, (b) V377F, (c) V441I and (d) G459S. Branch lengths are not drawn to scale. The amino acids for each interior and exterior node are shown.
(TIF)

Figure S3 **Nonsynonymous amino acid substitutions mapped onto the species topology of 23 mammals.** Branch lengths are not drawn to scale. Six sites on the ancestral branch of Old World fruit bats that had estimated omega values >1 are shown in red. Ancestral branches leading to Old World fruit bats and New World fruit bats are marked with blue and green lines, respectively.
(TIF)

Table S1 **The information of species examined in the study.**
(DOC)

Table S2 **The information of primers used for** ***Slc2a4*** **coding sequences amplification.**
(DOC)

Table S3 **Summarization of amino acid substitutions occurred on major branches.**
(DOC)
