# Supplementary figures and images for: Adaptive Evolution in the Glucose Transporter 4 Gene Slc2a4 in Old World Fruit Bats (Family: Pteropodidae)
Source: PLoS One. 2012 Apr 6;7(4):e33197. doi: 10.1371/journal.pone.0033197 (PMC3320886; doi:10.1371/journal.pone.0033197)

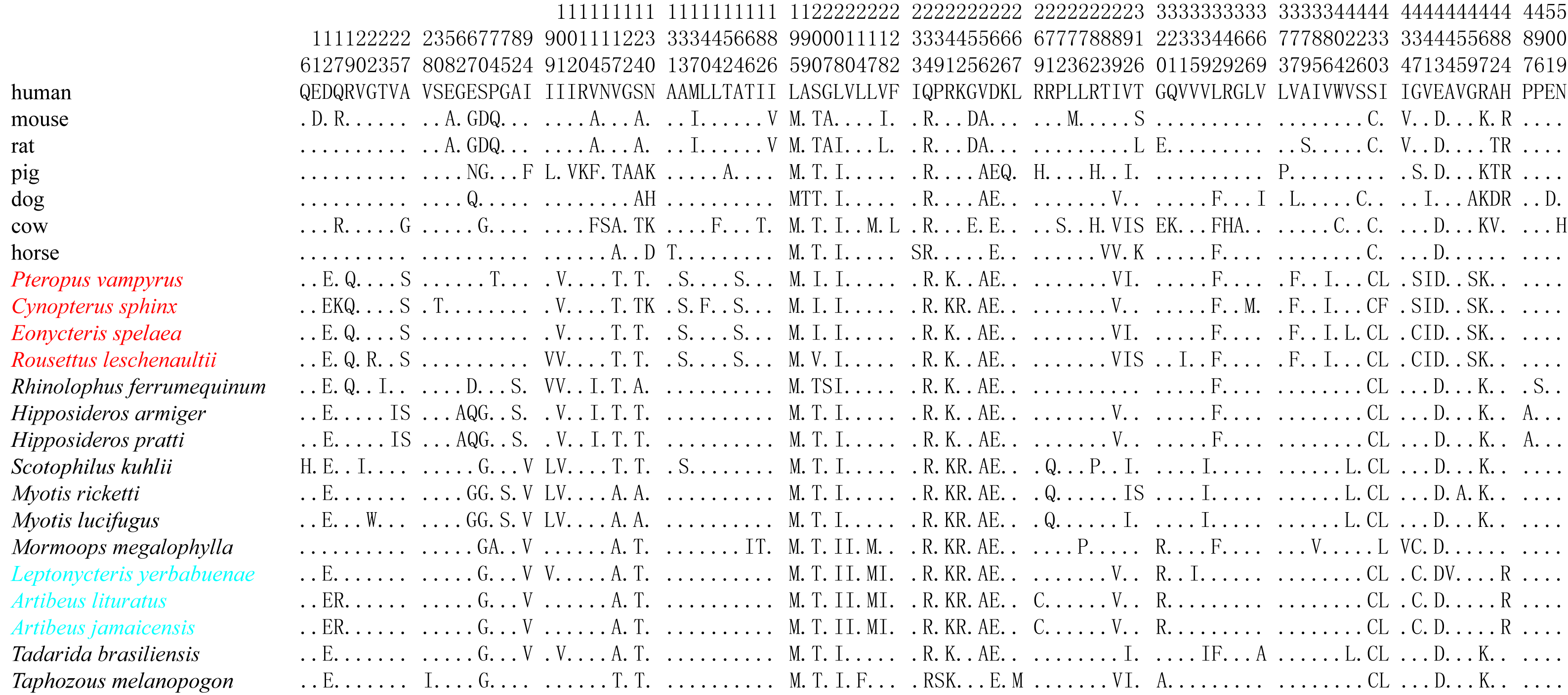

Supplement: Figure S1 — Alignment of the amino acid sequences of the Slc2a4 gene from 23 mammals (only the variable sites are shown). The species of Old World fruit bats and New World fruit bats are marked in red and blue, respectively. (TIF) [file pone.0033197.s001.tif]

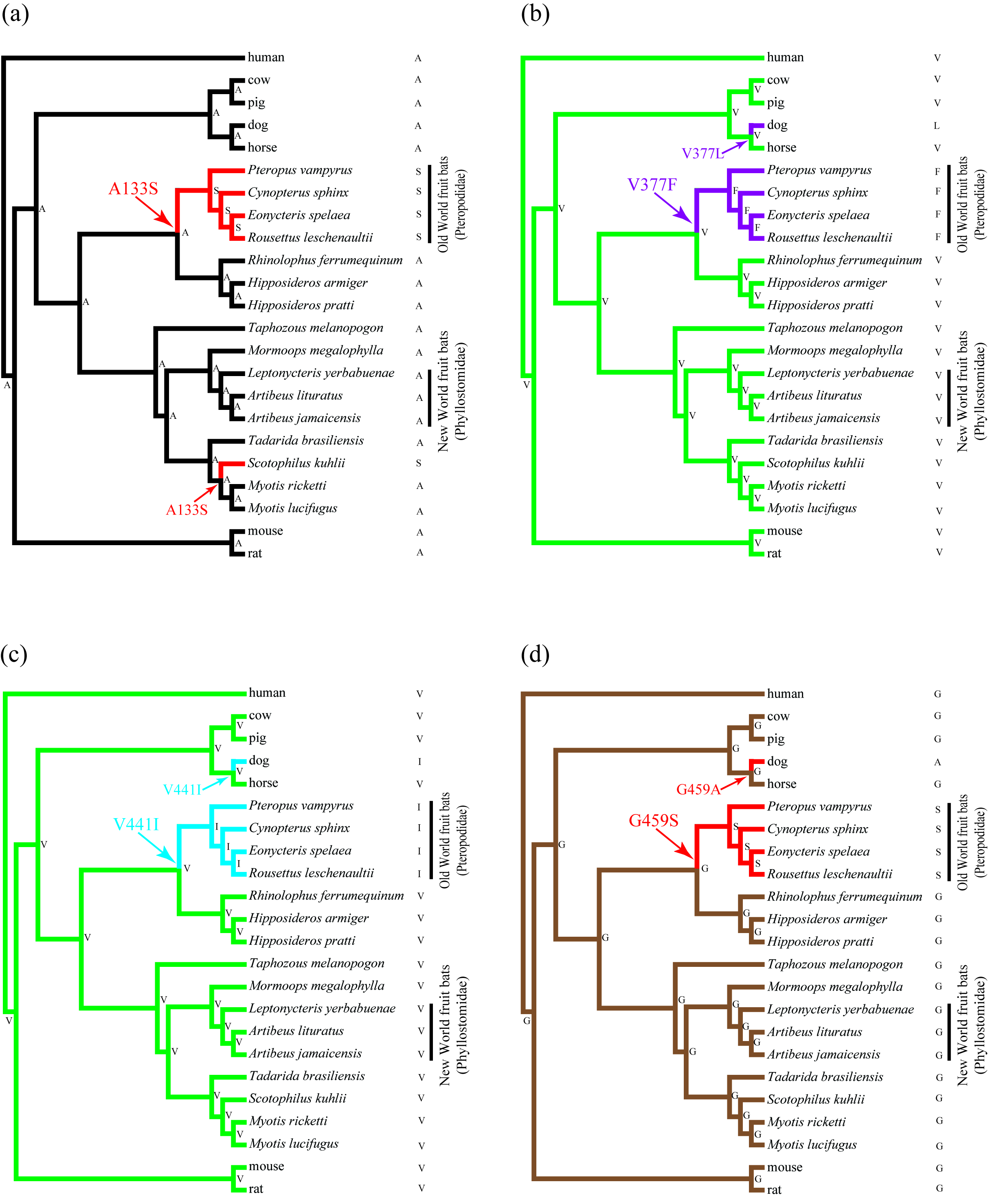

Supplement: Figure S2 — The species tree of 23 mammals with Old World fruit bats specific amino acid replacements were highlighted by ancestral sequence reconstruction using maximum parsimony method. (a) A133S, (b) V377F, (c) V441I and (d) G459S. Branch lengths are not drawn to scale. The amino acids for each interior and exterior node are shown. (TIF) [file pone.0033197.s002.tif]

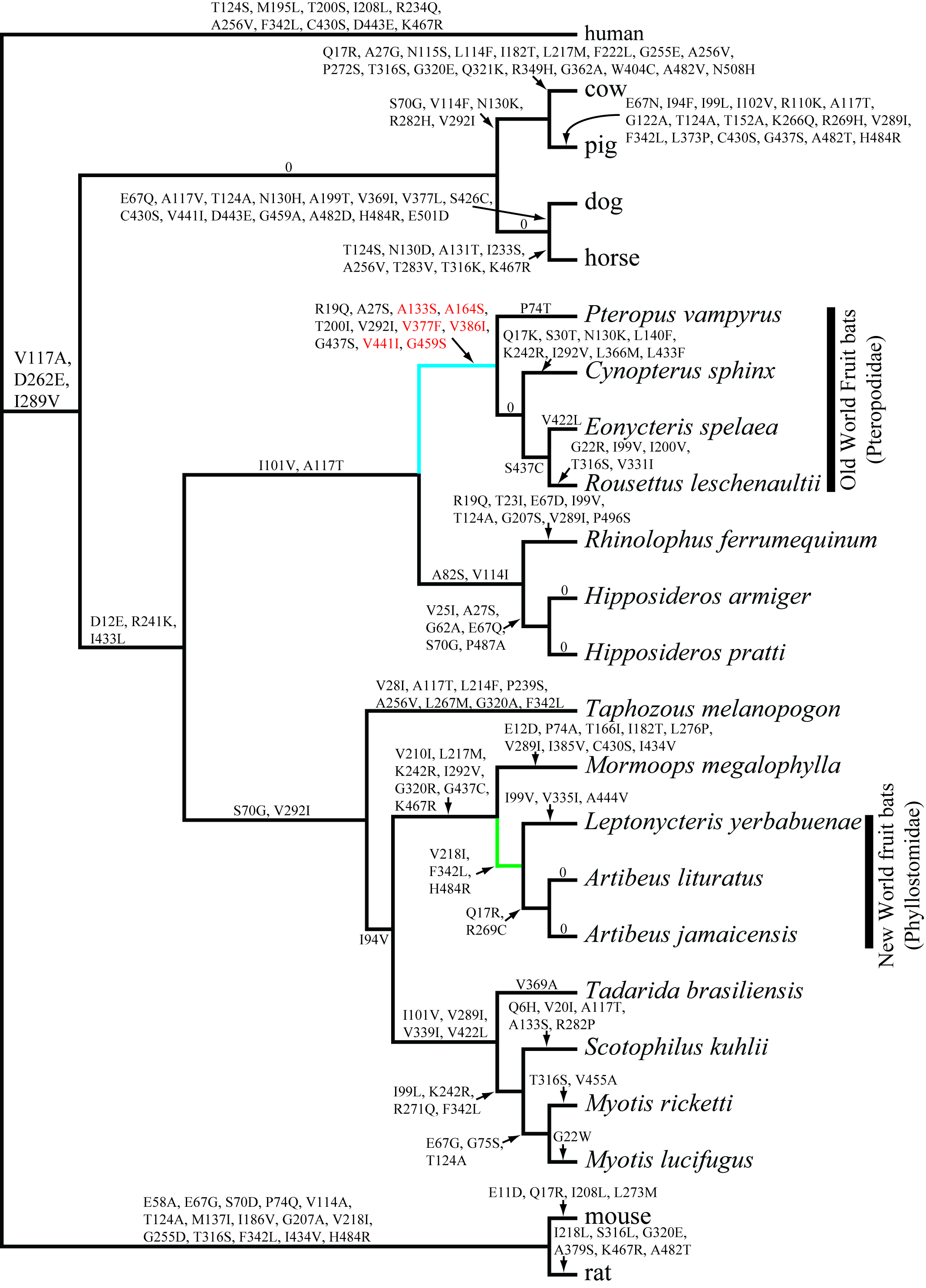

Supplement: Figure S3 — Nonsynonymous amino acid substitutions mapped onto the species topology of 23 mammals. Branch lengths are not drawn to scale. Six sites on the ancestral branch of Old World fruit bats that had estimated omega values >1 are shown in red. Ancestral branches leading to Old World fruit bats and New World fruit bats are marked with blue and green lines, respectively. (TIF) [file pone.0033197.s003.tif]
