# Supplementary material for: Adaptive Evolution in the Glucose Transporter 4 Gene Slc2a4 in Old World Fruit Bats (Family: Pteropodidae)
Source: PLoS One. 2012 Apr 6;7(4):e33197. doi: 10.1371/journal.pone.0033197 (PMC3320886; doi:10.1371/journal.pone.0033197)
Supplement: Table S1 — The information of species examined in the study. (DOC) [file pone.0033197.s004.doc]

**Table S1. The information of species examined in the study**

| **Species** | **Accession Number** | **Sampling Locality** |
| --- | --- | --- |
| *Pteropus vampyrus* | JN695660 | Indonesia |
| *Cynopterus sphinx* | JN695653 | China |
| *Eonycteris spelaea* | JN695654 | China |
| *Rousettus leschenaultii* | JN695662 | China |
| *Rhinolophus ferrumequinum* | JN695661 | China |
| *Hipposideros pratti* | JN695656 | China |
| *Hipposideros armiger* | JN695655 | China |
| *Taphozous melanopogon* | JN695665 | China |
| *Tadarida brasiliensis* | JN695664 | Mexico |
| *Myotis ricketti* | JN695659 | China |
| *Scotophilus kuhlii* | JN695663 | China |
| *Mormoops megalophylla* | JN695658 | Mexico |
| *Leptonycteris yerbabuenae* | JN695657 | Mexico |
| *Artibeus lituratus* | JN695652 | Mexico |
| *Artibeus jamaicensis* | JN695651 | Mexico |
| *Myotis lucifugus* | Ensembl database | — |
| *Homo sapiens* | NM_001042 | — |
| *Mus musculus* | NM_009204 | — |
| *Rattus norvegicus* | NM_012751 | — |
| *Equus caballus* | NM_001081866 | — |
| *Bos taurus* | NM_174604 | — |
| *Sus scrofa* | NM_001128433 | — |
| *Canis familiaris* | NM_001159327 | — |
