# Supplementary material for: Adaptive Evolution in the Glucose Transporter 4 Gene Slc2a4 in Old World Fruit Bats (Family: Pteropodidae)
Source: PLoS One. 2012 Apr 6;7(4):e33197. doi: 10.1371/journal.pone.0033197 (PMC3320886; doi:10.1371/journal.pone.0033197)
Supplement: Table S2 — The information of primers used for Slc2a4 coding sequences amplification. (DOC) [file pone.0033197.s005.doc]

**Table S2. The information of primers used for *Slc2a4* coding sequences amplification**

| **Species**^a^ | **Primers pairs for PCR** | | **Tm (°C)**^b^ |
| --- | --- | --- | --- |
| *Cynopterus sphinx*^*^  *Eonycteris spelaea*^*^  *Rousettus leschenaultii*^*^  *Myotis ricketti*^*^  *Scotophilus kuhlii*^*^  *Artibeus lituratus*^*^ | Forward primer: 5’-TGCGTCTCCAGTTCCTAAGACAAG-3’  Reverse primer: 5’-GGAAGAGAGGGTTAAAGTGCTGC-3’ | | 59 |
| *Rhinolophus ferrumequinum*^*^  *Hipposideros pratti*^*^  *Hipposideros armiger*^*^ | Forward primer: 5’-GTTCCTAAGACAAGATGCCGTCG-3’  Reverse primer: 5’-GGAAGAGAGGGTTAAAGTGCTGC-3’ | | 59 |
| *Mormoops megalophylla*  *Leptonycteris yerbabuenae*  *Tadarida brasiliensis*  *Taphozous melanopogon*  *Pteropus vampyrus* | Section 1  (exons 1~2) | Forward primer: 5’-TGTTCTTCCAGCTCTTAAGACAAG-3’ | 61 |
|  |  | Reverse primer: 5’-CTCATTGTAGCTCTGTTCAATCAC-3’ |  |
|  | Section 2  (exons 3~7) | Forward primer: 5’-ATGCCGTCGGGCTTCCAACAGAT-3’ | 66.1 |
|  |  | Reverse primer: 5’-AGATGACGCCAAGGAGAAAGGAGG-3’ |  |
|  | Section 3  (exons 8~9) | Forward primer: 5’-CTTGGCTCCTTGCAGTTTGGCTAC-3’ | 66 |
|  |  | Reverse primer: 5’-TTCAAGGCCGAGGGAATGGTATGG-3’ |  |
|  | Section 4  (exons 10~11) | Forward primer: 5’-TGATGACTGTGGCTCTGCTTCTGC-3’ | 64 |
|  |  | Reverse primer: 5’-CTGGGGAAGAGAGGGTTAAAGTGC-3’ |  |
| *Artibeus jamaicensis* | Section 1  (exons 1~2) | Forward primer: 5’-CCTGCCTCCAGCTCCTGAGACAAG-3’ | 59 |
|  |  | Reverse primer: 5’-ACCATGCCGCCCACAGAAAAGAT-3’ |  |
|  | Section 2  (exons 3~7) | Forward primer: 5’-ATGCCGTCGGGCTTCCAACAGAT-3’ | 66 |
|  |  | Reverse primer: 5’-AGATGACGCCAAGGAGAAAGGAGG-3’ |  |
|  | Section 3  (exons 8~9) | Forward primer: 5’-CTTGGCTCCTTGCAGTTTGGCTAC-3’ | 66 |
|  |  | Reverse primer: 5’-TTCAAGGCCGAGGGAATGGTATGG-3’ |  |
|  | Section 4  (exons 10~11) | Forward primer: 5’-TGATGACTGTGGCTCTGCTTCTGC-3’ | 64 |
|  |  | Reverse primer: 5’-CTGGGGAAGAGAGGGTTAAAGTGC-3’ |  |

^a^Asterisks (*) denote bat species of which *Slc2a4* coding sequences were amplified from pectoral muscle cDNA. While for other bat species, *Slc2a4* coding sequences were amplified from genomic DNA.

^b^Tm, annealing temperature.
