# Supplementary material for: Adaptive Evolution in the Glucose Transporter 4 Gene Slc2a4 in Old World Fruit Bats (Family: Pteropodidae)
Source: PLoS One. 2012 Apr 6;7(4):e33197. doi: 10.1371/journal.pone.0033197 (PMC3320886; doi:10.1371/journal.pone.0033197)
Supplement: Table S3 — Summarization of amino acid substitutions occurred on major branches. (DOC) [file pone.0033197.s006.doc]

**Table S3. Summarization of amino acid substitutions occurred on major branches**

| **Mammal Branches** | **Amino Acid Substitutions^*^** |
| --- | --- |
| Human | T124S, M195L, T200S, I208L, R234Q, A256V, F342L, C430S, D443E, K467R |
| Cow | Q17R, A27G, N115S, L114F, I182T, L217M, F222L, G255E, A256V, P272S, T316S, G320E, Q321K, R349H, G362A, W404C, A482V, N508H |
| Pig | E67N, I94F, I99L, I102V, R110K, A117T, G122A, T124A, T152A, K266Q, R269H, V289I, F342L, L373P, C430S, G437S, A482T, H484R |
| Dog | E67Q, A117V, T124A, N130H, A199T, V369I, V377L, S426C, C430S, V441I, D443E, G459A, A482D, H484R, E510D |
| Horse | T124S, N130D, A131T, I233S, A256V, T283V, T316K, K467R |
| Ancestral branch of Rodents | E58A, E67G, S70D, P74Q, V114A, T124A, M137I, I186V, G207A, V218I, G255D, T316S, F342L, I434V, H484R |
| Mouse | E11D, Q17R, I208L, L273M |
| Rat | I218L, S316L, G320E, A379S, K467R, A482T |
| Chiroptera (bats) | D12E, R241K, I433L |
| Yinpterochiroptera | I101V, A117T |
| Yangochiroptera | S70G, V292I |
| Old World Fruit Bats | R19Q, A27S, A133S, A164S, T200I, V292I, V377F, V386I, G437S,V441I, G459S |
| New World Fruit Bats | V218I, F342L, H484R |
| *Rhinolophus ferrumequinum* | R19Q, T23I, E67D, I99V, T124A, G207S, V289I, P496S |
| *Hipposideros* | V25I, A27S, G62A, E67Q, S70G, P487A |
| *Taphozous melanopogon* | V28I, A117T, L214F, P239S, A256V, L267M, G320A, F342L |
| *Mormoops megalophylla* | E12D, P74A, T166I, I182T, L276P, V289I, I385V, C430S, I434V |
| *Scotophilus kuhlii* | Q6H, V20I, A117T, A133S, R282P |

^*^Amino acids changes to Serine (S) or Isoleucine (I) are underlined and colored red and blue, respectively.
